# Oral delivery of lycopene-loaded microemulsion for brain-targeting: preparation, characterization, pharmacokinetic evaluation and tissue distribution

**DOI:** 10.1080/10717544.2019.1689312

**Published:** 2019-11-18

**Authors:** Yunliang Guo, Xuyan Mao, Jing Zhang, Peng Sun, Haiyang Wang, Yue Zhang, Yingjuan Ma, Song Xu, Renjun Lv, Xueping Liu

**Affiliations:** a Department of Geriatrics, Shandong Provincial Hospital Affiliated to Shandong University, Jinan, PR China;; b Department of Geriatric Neurology, Shandong Provincial Hospital Affiliated to Shandong University, Jinan, PR China;; c Anti-Aging Monitoring Laboratory, Shandong Provincial Hospital Affiliated to Shandong University, Jinan, PR China;; d Bio-nano & Medical Engineering Institute, Jining Medical University, Jining, PR China;; e Department of Cell and Neurobiology, School of Basic Medical Sciences, Shandong University, Jinan, PR China;; f Institute of Materia Medica, Shandong Academy of Medical Sciences, Jinan, PR China;; g Shandong Provincial Hospital, Shandong First Medical University & Shandong Academy of Medical Sciences, Jinan, PR China;; h Department of Anti-Aging, Shandong Provincial Hospital Affiliated to Shandong University, Jinan, PR China

**Keywords:** Lycopene, microemulsion, pharmacokinetic study, tissue distribution, brain-targeting

## Abstract

Lycopene is considered as a promising neuroprotector with multiple bioactivities, while its therapeutic use in neurological disorders is restricted due to low solubility, instability and limited bioavailability. Our work aimed to develop lycopene-loaded microemulsion (LME) and investigate its potentials in improving bioavailability and brain-targeting efficiency following oral administration. The blank microemulsion (ME) excipients were selected based on orthogonal design and pseudo-ternary phase diagrams, and LME was prepared using the water titration method and characterized in terms of stability, droplet size distribution, zeta potential, shape and lycopene content. The optimized LME encompassed lycopene, (*R*)-(+)-limonene, Tween 80, Transcutol HP and water and lycopene content was 463.03 ± 8.96 µg/mL. This novel formulation displayed transparent appearance and satisfactory physical and chemical stabilities. It was spherical and uniform in morphology with an average droplet size of 12.61 ± 0.46 nm and a polydispersity index (PDI) of 0.086 ± 0.028. The pharmacokinetics and tissue distributions of optimized LME were evaluated in rats and mice, respectively. The pharmacokinetic study revealed a dramatic 2.10-fold enhancement of relative bioavailability with LME against the control lycopene dissolved in olive oil (LOO) dosage form in rats. Moreover, LME showed a preferential targeting distribution of lycopene toward brain in mice, with the value of drug targeting index (DTI) up to 3.45. In conclusion, the optimized LME system demonstrated excellent physicochemical properties, enhanced oral bioavailability and superior brain-targeting capability. These findings provide a basis for the applications of ME-based strategy in brain-targeted delivery via oral route, especially for poorly water-soluble drugs.

## 1. Introduction

Lycopene, a pigment belonging to carotenoid family, mostly exists in tomatoes and other fruits with red color (Gerster, 1997). Due to highly polyunsaturated hydrocarbons, lycopene serves as an efficient antioxidant and singlet-oxygen quencher, and has demonstrated diverse and remarkable bioactivities, such as anti-oxidative stress (Kaur et al., 2011), anti-inflammation (Palozza et al., 2011), anti-apoptosis (Fujita et al., 2013) and anti-cancer (Ilic & Misso, 2012). Moreover, lycopene can pass through the blood–brain barrier (BBB) into central nervous system and exert neuroprotective effects against neurological disorders (Kaur et al., 2011; Liu et al., 2018).

Nevertheless, the potential pharmacological use of lycopene for neurological disease treatment in clinical practice still remains questionable. Lycopene is almost insoluble in water, and the solubility is rather low in most kinds of oils. From the configurational point of view, multiple linear conjugated double bonds make it susceptible to degradation when exposed to oxygen, sunlight, heat, acid or metal ions (Lee & Chen, 2002). Additionally, the absolute bioavailability of lycopene was found to be extremely low, with 1.85 ± 0.39% in an experimental rat model (Faisal et al., 2010). Therefore, it is in urgent need to develop novel dosage form for lycopene to elevate solubility and stability, as well as improving oral bioavailability and further brain efficacy.

Microemulsion (ME) is a transparent colloidal system mixed by oil, surfactant, co-surfactant and water (Lawrence & Rees, 2000; Karasulu, 2008). The spontaneously formed dispersion is optically isotropic and thermodynamically stable, with droplet size less than 100 nm (Karasulu, 2008). Owing to manifold advantages of slight materials requirement, easy manufacture, smaller size and monodispersibility, ME has been extensively used to deliver drugs (Ghosh et al., 2006; Sane et al., 2013). More importantly, it is known to have higher solubilization capacity, especially for hydrophobic substances (Amar et al., 2003; Chen et al., 2017), and the system possesses promising stability to protect bioactive components from undesired damage (Chen et al., 2017; Tao et al., 2017).

Among different delivery systems, ME has been successfully utilized to enhance oral bioavailability of poorly water-soluble compounds (Kawakami et al., 2002a; Araya et al., 2005; Ghosh et al., 2006). The improvement of oral bioavailability is due to either individual or a combination of multiple factors, such as greater solubilization, absorption enhancement, as well as modified permeability and metabolism profiles (Yin et al., 2009; Mohsin et al., 2016; Subongkot & Ngawhirunpat, 2017). Moreover, the BBB restricts access of various drugs into the brain, which compromises therapeutic efficacy (Henderson & Piquette-Miller, 2015). Current evidence shows that ME could be applied to promote targeted drug delivery to the brain (Ma et al., 2013; Shinde & Devarajan, 2017; Yi et al., 2017). The lipid-based formulations and nano-sized particles make it more efficiently to cross the BBB (Shah et al., 2018). Hence, the ME system becomes an attractive choice for lycopene oral delivery, particularly to brain.

However, to the best of our knowledge, there is no ME-based strategy applied to enhance oral bioavailability or brain-targeting efficiency of lycopene. In the present investigation, it was hypothesized that oral delivery of lycopene-loaded microemulsion (LME) could improve bioavailability and enhance biodistribution of lycopene in the brain. Thus, the objectives of this study were to: (1) select excipients and prepare LME; (2) characterize and optimize LME; (3) assess the impact of optimized LME on the pharmacokinetics in rats by high-performance liquid chromatography (HPLC) method and (4) evaluate tissue distributions and brain-targeting parameters of optimized LME in mice.

## 2. Materials and methods

### 2.1. Substances and reagents

Lycopene (Batch No. KS170313, 98.06% purity) was purchased from Shaanxi Kingsci Biotechnology Co., Ltd. (Shaanxi, China). The internal standard retinyl acetate (98.4% purity) was obtained from Shanghai Huicheng Biological Technology Co., Ltd. (Shanghai, China). Tween 80 (polysorbate 80, injection grade) was acquired from Nanjing Well Chemical Co., Ltd. (Nanjing, China). Transcutol HP (designed for oral administration, 99.986% purity) was received as gratis sample from Gattefossé (Lyon, France) and polyethylene glycol 400 (PEG 400) was provided by Tianjin Dingshengxin Chemical Industry Co., Ltd. (Tianjin, China). (*R*)-(+)-Limonene (97% purity), olive oil, glycerol, butylated hydroxytoluene (BHT), ethyl acetate, anhydrous ethanol and hexane were all bought from Shanghai Macklin Biochemical Technology Co., Ltd. (Shanghai, China). Ethyl oleate and oleic acid were obtained from Tianjin Guangfu Fine Chemical Research Institute (Tianjin, China) and Damao Chemical Reagent Factory (Tianjin, China), respectively. Soybean oil (medical grade) was procured from Jiangxi Yipusheng Pharmaceutical Co., Ltd. (Jiangxi, China). Corn oil was supplied by Shanghai Yuanye Biological Technology Co., Ltd. (Shanghai, China). Deionized water was generated with an ELGA-Purelab water purification system (Model CLXXUVFM2; ELGA LabWater, High Wycombe, UK). The HPLC grade mobile phase components methanol and acetonitrile were from TEDIA Company, Inc. (Fairfield, OH, USA), while dichloromethane was from Tianjin Kemio Chemical Reagent Exploitation Center (Tianjin, China). The centrifugal ultrafilter (0.22 µm pore size) was purchased from Millipore (Bedford, MA, USA). All other reagents used in the study were of analytical grade.

### 2.2. Animals

Wistar rats and C57BL/6 mice were supplied from the Laboratory Animal Center of Shandong University and Jinan Pengyue Experimental Animal Breeding Co., Ltd. (Jinan, China), respectively. Rats and mice were kept under standard laboratory conditions for 7 days prior to use in the Experimental Animal Center of Shandong Provincial Hospital, and were prohibited from eating foods containing lycopene. All animal experiments complied with the requirements of the National Act on the Use of Experimental Animals (People’s Republic of China) and were conducted using protocols approved by the Animal Care and Utilization Committee of Shandong Provincial Hospital Affiliated to Shandong University.

### 2.3. HPLC analysis

Lycopene was assayed by HPLC (Thermo Dionex UltiMate 3000 liquid chromatography systems, Thermo Fisher Scientific, Waltham, MA, USA) using a Thermo Hypersil Gold C18 column (250 mm × 4.6 mm, 5 µm, Thermo Fisher Scientific), with column temperature at 25 °C. The mobile phase consisted of a mixture of methanol, acetonitrile and dichloromethane (50:33:17, v/v/v) delivered at a flow rate of 1.0 mL/min. The diode array detection wavelength was set at 472 nm and the injection volume was 10 µL. The chromatographic conditions were applied throughout this study.

Lycopene concentration was obtained from standard curve. Stock solutions of lycopene were prepared by dissolving accurately weighed standard compounds in ethyl acetate, and serially diluted working solutions were obtained through stepwise dilutions of the stock solution with mobile phase, then the standard curve was yielded.

### 2.4. Screening of ME compositions

The selection of oil was performed by saturated solubility study. An excess amount of lycopene was added individually to various oils in lightproof glass vials flushed with nitrogen gas. Mixtures were magnetically stirred for 24 h and maintained at 25 °C, followed by centrifugation at 13,000 rpm for 10 min to remove the excess lycopene. The supernatant was then filtered through a 0.22 µm membrane after which lycopene concentration in the supernatant was determined using HPLC after a suitable dilution with mobile phase. Solubility studies were conducted in triplicate and oils with higher lycopene solubility were selected for optimization.

The screenings of surfactant, co-surfactant and surfactant to co-surfactant ratio (surfactant/co-surfactant, w/w) were carried out, respectively. Tween 80, widely employed as surfactant in pharmaceutical studies, was selected for its reported property of superior emulsifying capacity, appropriate hydrophilic-lipophilic balance value (HLB = 15) and enhanced brain-targeting (Sun et al., 2004; Craparo et al., 2008). Transcutol HP, PEG 400 and glycerol were commonly applied as co-surfactants due to high biocompatibility and safety, so they were chosen for further investigations. Additionally, surfactant to co-surfactant ratios of 2:1, 3:2 and 3:1 were used for selection during follow-up experiments to achieve stronger interactions between surfactant and co-surfactant.

### 2.5. Orthogonal optimization and construction of pseudo-ternary phase diagrams

The oil, co-surfactant and surfactant to co-surfactant ratio were selected as three factors affecting ME formation, each containing three levels (Supplementary Table S1), and the standard L_9_ (3^4^) orthogonal design was used for optimization and further analysis (see Table 2).

Pseudo-ternary phase diagrams were elaborated to find out the optimal compositions of blank ME (without lycopene) by the water titration method at 25 °C. The surfactant (Tween 80) and different co-surfactants (Transcutol HP, PEG 400 and glycerol) were mixed at various ratios (2:1, 3:2 and 3:1) according to orthogonal design to make the surfactant and co-surfactant mixture (S_mix_). Afterwards, selected oil phases and S_mix_ were mixed homogeneously under continuous stirring for 30 min to obtain corresponding clear oily mixtures, where the ratios of oil to S_mix_ were varied from 9:1 to 1:9 (w/w). Then distilled water was added dropwise to each oily mixture with moderate stirring to make it well-equilibrated, and the amount of water was recorded when transparency-to-turbidity transition occurred. Pseudo-ternary phase diagrams were constructed using OriginPro 8.5 software. Samples with a transparent appearance during titration were defined as the ME region within phase diagrams, and optimum combinations and ratios were determined on the basis of orthogonal design and areas of ME region.

### 2.6. Preparation of LME

Based on the results of orthogonal optimizing experiments and pseudo-ternary phase diagrams, the optimal compositions of blank ME were finalized (oil: (*R*)-(+)-limonene; co-surfactant: Transcutol HP; surfactant to co-surfactant ratio: 2:1, w/w). Our preliminary experiments also revealed that the blank ME possessed superior stability when the ratios of oil to S_mix_ were set at 1:9 and 2:8 (w/w), while not for the remaining ratios during centrifugation after storage for 1 week. Thus, several ME formulations with incorporation of lycopene were selected for further characterization and optimization (Table 3).

In order to obtain the maximal loading content of lycopene in ME, an excess amount of lycopene was dissolved into the optimum oil phase ((*R*)-(+)-limonene) in light-proof containers flushed with nitrogen gas by vortexing for 5 min, after which LME was prepared as mentioned above. It was then magnetically stirred for 24 h at 25 °C. The undissolved lycopene was removed by centrifugation at 13,000 rpm for 10 min, followed by filtering through a 0.22 µm membrane to yield LME.

### 2.7. Characterization of LME formulations

#### 2.7.1. Physical stability

The transparency of LME appearance was determined by visual inspection. After 1 week of storage at 25 °C, the physical stability was evaluated by observing precipitation, creaming and cracking and LME formulations were subjected to centrifugation at 10,000 rpm for 30 min and observed for phase separation.

#### 2.7.2. Droplet size distribution and zeta potential

The average droplet size, polydispersity index (PDI) and zeta potential of LME formulations were measured using dynamic light scattering (DLS) analyzers, of which average droplet size and PDI were determined by Zetasizer Nano ZS (Malvern instruments, Worcestershire, UK), while zeta potential was evaluated by Delsa Nano (Beckman Coulter instruments, Brea, CA, USA). The determinations of droplet size distribution and zeta potential were conducted by taking appropriate volume of samples into quartz cuvettes and quartz capillary cells at 25 °C, all in triplicates, respectively.

#### 2.7.3. Drug content determination

After equilibration, the samples were diluted with an appropriate amount of mobile phase, and lycopene content was determined with previously developed HPLC method for triplicate.

#### 2.7.4. Morphological evaluation

The morphology of optimized LME was observed by transmission electron microscopy (TEM, JEM-1200, Tokyo, Japan). In brief, a drop of diluted ME sample was deposited onto a film-coated copper grid, and the excess sample volume was removed with a filter paper. The sample was then negatively stained by a drop of 2% phosphotungstic acid solution, and was allowed to dry at room temperature before TEM imaging.

### 2.8. Stability study during storage

The optimized LME and same content of lycopene dissolved in olive oil (LOO) were prepared and stored in light-protected and tightly sealed containers flushed with nitrogen gas at 4 and 25 °C, respectively. After 0 day as well as 1, 2, 4, 6 and 8 weeks of storage, both the physical and chemical stabilities were assessed. The physical stability evaluations of LME included visualization of clarity and observation of precipitation after centrifugation at 10,000 rpm for 30 min. The chemical stability was evaluated by determining the remaining lycopene content using HPLC. The percentage of lycopene remaining was compared with the amount on day 0 (baseline). The samples were determined and analyzed in triplicate.

### 2.9. Pharmacokinetic study

#### 2.9.1. Rats and treatments

The pharmacokinetic study of the test group (optimized LME) and control group (LOO) was performed in male Wistar rats (weighing 255 ± 5 g). Prior to experiments, a total of 12 rats were prohibited from feeding for 12 h with free access to water, and were randomly divided into two groups (*n* = 6 in each group). They were orally administered two dosage forms separately at a dose of 8 mg/kg based on lycopene concentration. At 0.25, 0.5, 1, 2, 4, 6, 8, 12, 24 and 48 h after administration, 0.5 mL of blood sample was collected from the jugular vein and poured into a heparinized tube. All rats remained healthy after blood collection for 10 time points. Then blood samples were immediately transferred to ice bath, followed by centrifugation at 4000 rpm for 10 min (4 °C) to obtain plasma. The plasma samples were prevented from light exposure and flushed with nitrogen gas for storage at −80 °C until analysis.

#### 2.9.2. Analysis of rat plasma samples

A validated method reported by Talwar et al. (1998) was modified to extract lycopene from rat plasma. Briefly, 100 µL of plasma sample was pretreated by precipitating protein using 100 µL of anhydrous ethanol, and lycopene was extracted with 200 µL of hexane (containing 0.01% BHT). BHT was added to prevent oxidation of lycopene during extraction. The mixture was vortexed for 1 min and centrifuged at 4000 rpm for 6 min to collect supernatant. The bottom layer was repeatedly extracted, and all the supernatants were combined and evaporated to dryness under nitrogen gas. The residue was then reconstituted in 100 µL of dichloromethane, followed by centrifugation at 4000 rpm for 5 min with a 0.22 µm centrifugal ultrafilter, and the filtered sample was used for HPLC analysis.

Plasma calibration standards were obtained by dilution of the corresponding working solutions with blank rat plasma, so that the standard curve of rat plasma could be prepared.

### 2.10. Tissue distribution study

#### 2.10.1. Mice and treatments

Male C57BL/6 mice (25 ± 2 g) were fasted 12 h and were randomly assigned to two groups (*n* = 48 in each group). The test (optimized LME) and control (LOO) dosage forms were administered orally at a single lycopene dose of 8 mg/kg. At predetermined times (0.5, 1, 3, 6, 9, 12, 24 and 48 h) after administration, blood was collected from six mice at every time point in each group, respectively. The separation and storage of mouse plasma samples were same as rat plasma. Thereafter, mice were humanely sacrificed, then tissues (brain, heart, liver, spleen, lung and kidney) were promptly harvested, washed with ice-cold saline and dried with filter paper. Tissue samples were packed and stored in vacuum bags separately at −80 °C for further analysis.

#### 2.10.2. Analysis of mouse tissue samples

Mouse plasma samples were handled just as rat plasma, while other tissue samples were weighed and homogenized with an equal aliquot of normal saline to obtain tissue homogenate (0.5 g/mL, w/v). Afterwards, 500 µL (for brain, liver and kidney) or 200 µL (for heart, spleen and lung) of tissue homogenate was mixed with identical volumes of anhydrous ethanol for protein precipitation and hexane (containing 0.01% BHT) for lycopene extraction, respectively, and the following steps were performed according to the protocol described in the section ‘Analysis of rat plasma samples’. The concentrations of lycopene in mouse tissues were also determined by HPLC.

Calibration standards were prepared by spiking blank mouse plasma or homogenate of different tissues with multiple concentrations of working solutions, and the corresponding standard curves were made.

### 2.11. Analytical method validation

Method validation was performed according to the modified protocols proposed by Talwar et al. (1998) prior to the determination of collected samples. No interference of endogenous compounds was observed for all plasma and tissue samples under the chromatographic conditions used, indicating the specificity of the method. In addition, the intra-day and inter-day precisions, accuracy, extraction recovery and sample stability were assessed by analyzing quality control samples at three different concentrations (low, medium and high) in rat plasma, together with mouse plasma, brain and liver.

### 2.12. Data analysis and statistics

The pharmacokinetic parameters of plasma and tissues were all calculated by non-compartmental analysis on the basis of statistical moment theory using the Drug and Statistics (DAS) software 2.0 (Chinese Pharmacological Society), including area under the concentration–time curve (AUC), peak concentration (*C*_max_), time to reach peak concentration (*T*_max_), half-life (*t*_1/2_), mean residence time (MRT) and plasma clearance (CL).

In rats, the relative bioavailability of oral administration was described in Equation (1):

(1) $$\text{Relative}\;\text{bioavailability}\;\left(\%\right)=\frac{{(\text{AUC})}_{\text{LME}}}{{(\text{AUC})}_{\text{LOO}}}\times 100\%.$$

In mice, the tissue targeting efficiency was evaluated by corresponding parameters, including the relative rates of uptake (*R*e) and the ratio of peak concentration (*C*e), which were calculated using Equations (2) and (3):

(2) $$Re=\frac{{(\text{AUC})}_{\text{tissue}\;\text{of}\;\text{LME}}}{{(\text{AUC})}_{\text{tissue}\;\text{of}\;\text{LOO}}},$$

(3) $$Ce=\frac{{(C_{\text{max}})}_{\text{tissue}\;\text{of}\;\text{LME}}}{{(C_{\text{max}})}_{\text{tissue}\;\text{of}\;\text{LOO}}},$$

where (AUC)_tissue_ denotes area under the concentration–time curve in one tissue.

In order to better assess blood-to-tissue direct transport, we introduced the term of drug targeting index (DTI) (Ren et al., 2013), and the equation was as follows (Equation (4)):

(4) $$\text{DTI}=\frac{{(\text{AUC})}_{\text{tissue}\;\text{of}\;\text{LME}}/{(\text{AUC})}_{\text{plasma}\;\text{of}\;\text{LME}}}{{(\text{AUC})}_{\text{tissue}\;\text{of}\;\text{LOO}}/{(\text{AUC})}_{\text{plasma}\;\text{of}\;\text{LOO}}},$$

where (AUC)_tissue_ and (AUC)_plasma_ represent area under the concentration–time curve determined in tissues and plasma of mice, respectively, and DTI >1 was considered as the targeting distribution.

The results were presented as mean ± standard deviation (SD). Measurement data were analyzed by one-way or two-way analysis of variance (ANOVA) to determine the intergroup differences, while for enumeration data, the Mann–Whitney U-test was used. Statistical calculations were carried out using SPSS statistics software 23.0 (SPSS Inc., Chicago, IL, USA), and all results were considered to be significant at *p* < .05.

## 3. Results

### 3.1. Solubility study

The solubility profile of lycopene in various oils is presented in Table 1. (*R*)-(+)-Limonene (3.06 ± 0.18 mg/mL) demonstrated the highest solubilization capacity among different oils, followed by ethyl oleate (1.76 ± 0.21 mg/mL) and oleic acid (1.45 ± 0.13 mg/mL), so they were chosen for orthogonal optimization with the goals of increasing lycopene solubility and better assessing their compatibility with other constituents.

**Table 1.** Solubility of lycopene in various oils at 25 °C.

Oils	Solubility (mg/mL)
(*R*)-(+)-Limonene	3.06 ± 0.18
Ethyl oleate	1.76 ± 0.21
Oleic acid	1.45 ± 0.13
Olive oil	1.15 ± 0.25
Soybean oil	0.64 ± 0.19
Corn oil	0.90 ± 0.40

Each value is the mean ± SD of three separate determinations.

### 3.2. Orthogonal optimization and construction of pseudo-ternary phase diagrams

**Figure 1.**
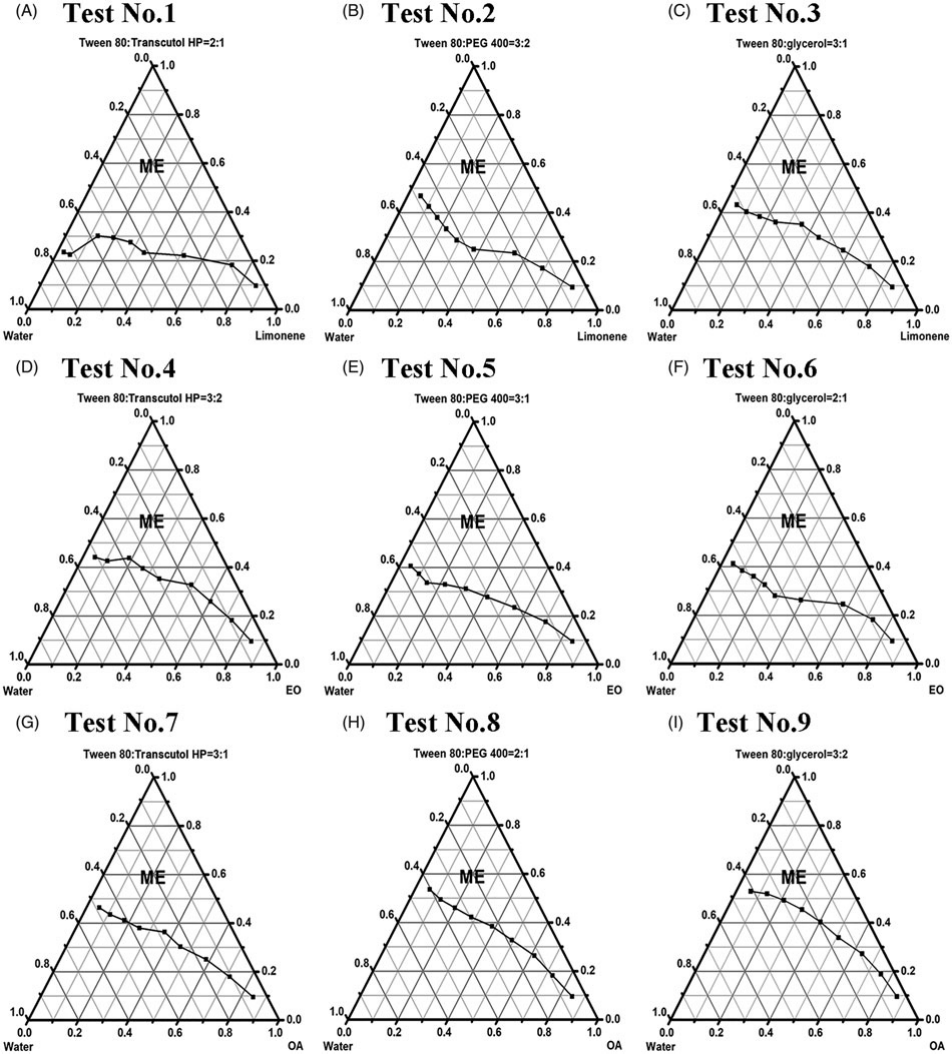
Construction of pseudo-ternary phase diagrams based on orthogonal design at 25 °C. (A) Test No.1: limonene (oil), S_mix_ (Tween 80/Transcutol HP = 2:1, w/w) and water; (B) test No.2: limonene (oil), S_mix_ (Tween 80/PEG 400 = 3:2, w/w) and water; (C) test No.3: limonene (oil), S_mix_ (Tween 80/glycerol = 3:1, w/w) and water; (D) EO (oil), S_mix_ (Tween 80/Transcutol HP = 3:2, w/w) and water; (E) EO (oil), S_mix_ (Tween 80/PEG 400 = 3:1, w/w) and water; (F) EO (oil), S_mix_ (Tween 80/glycerol = 2:1, w/w) and water; (G) OA (oil), S_mix_ (Tween 80/Transcutol HP = 3:1, w/w) and water; (H) OA (oil), S_mix_ (Tween 80/PEG 400 = 2:1, w/w) and water; (I) OA (oil), S_mix_ (Tween 80/glycerol = 3:2, w/w) and water. The region of blank microemulsion (without lycopene) is labeled ME. Limonene: (*R*)-(+)-limonene; EO: ethyl oleate; OA: oleic acid; S_mix_: the surfactant and co-surfactant mixture.

The design of orthogonal experiments is displayed in Supplementary Table S1 and Table 2. Figure 1 depicts the constructed pseudo-ternary phase diagrams, and calculated areas of ME region are shown in Table 2.

**Table 2.** The design and results of orthogonal optimizing experiments.

Test no.	Factors			D	Areas of ME region
	A (oil)	B (co-surfactant)	C (surfactant/co-surfactant)		
1	1	1	1	1	60.0
2	1	2	2	2	51.5
3	1	3	3	3	48.0
4	2	1	2	3	43.5
5	2	2	3	1	53.5
6	2	3	1	2	52.0
7	3	1	3	2	45.5
8	3	2	1	3	39.0
9	3	3	2	1	34.5
Mean 1	53.2	49.7	50.3	49.3	
Mean 2	49.7	48.0	43.2	49.7	
Mean 3	39.7	44.8	49.0	43.5	
Range	13.5	4.9	7.1	6.2	

ME: microemulsion.

The three levels (1, 2 and 3) of factors are shown in Supplementary Table S1. Based on the standard L_9_ (3^4^) orthogonal design, D indicates blank column. The area of ME region was employed as evaluation index. Calculation of ME region area: the area of the smallest triangle in pseudo-ternary phase diagrams is defined as 1, and the number of triangles in ME region is counted (accurate to 0.5). Range denotes the difference between maximum mean and minimum mean among three levels (1, 2 and 3). Surfactant/co-surfactant denotes the surfactant to co-surfactant ratio (w/w).

As exhibited in Table 2, the ranges were in the order of oil > surfactant to co-surfactant ratio > co-surfactant, suggesting that oil phase played the most important role in ME formation and region area, followed by surfactant to co-surfactant ratio and co-surfactant. Besides, due to the maximal means of level 1 in all three factors, the combination of A1B1C1, that is, with (*R*)-(+)-limonene as oil, Transcutol HP as co-surfactant and surfactant (Tween 80) to co-surfactant ratio = 2:1 (w/w), was considered to be optimal for blank ME preparation (see Figure 1(A)).

### 3.3. Preparation and characterization of LME formulations

In the procedure of blank ME preparation, we found that when the ratios of oil ((*R*)-(+)-limonene) to S_mix_ (Tween 80: Transcutol HP = 2:1, w/w) were fixed at 1:9 and 2:8 (w/w), the system’s stability is superior during storage and centrifugation. However, when the ratios varied from 3:7 to 9:1 (w/w), a little precipitation was observed when samples were subjected to centrifugation after storage for 1 week at 25 °C. Therefore, some samples of LME with oil to S_mix_ ratios of 1:9 (w/w) (LME 1 − 3) or 2:8 (w/w) (LME 4 − 6) were prepared and characterized in terms of various parameters for optimization (Table 3).

**Table 3.** Composition and characterization of different LME formulations.

Formulations	Composition (%, w/w)			Average droplet size (nm)	PDI	Zeta potential (mV)	Lycopene content (µg/mL)
	Oil^a^	S_mix_^b^	Water				
LME 1	5	45	50	17.15 ± 2.16	0.501 ± 0.017	−0.16 ± 0.03	297.47 ± 23.71
LME 2	4	36	60	12.41 ± 0.18	0.079 ± 0.030	−0.29 ± 0.06	257.21 ± 27.00
LME 3	3	27	70	12.27 ± 0.53	0.066 ± 0.011	−0.44 ± 0.10	173.44 ± 14.10
LME 4	10	40	50	37.66 ± 20.61	0.456 ± 0.108	−0.22 ± 0.07	606.09 ± 17.69
LME 5	8	32	60	12.61 ± 0.46	0.086 ± 0.028	−0.49 ± 0.12	463.03 ± 8.96
LME 6	6	24	70	12.85 ± 0.53	0.076 ± 0.021	−0.46 ± 0.26	321.71 ± 11.85

S_mix_: the surfactant and co-surfactant mixture; LME: lycopene-loaded microemulsion; PDI: polydispersity index.

Results are presented as mean ± SD (*n* = 3).

a (*R*)-(+)-Limonene and lycopene.

b Surfactant (Tween 80) to co-surfactant (Transcutol HP) ratio = 2:1, w/w.

The optical appearance of LME formulations (LME 1 − 6) was transparent. After 1 week of storage at 25 °C, no precipitation, creaming or cracking was found for all samples, and no phase separation was observed upon centrifugation at 10,000 rpm for 30 min either, indicating superior physical stability.

**Figure 2.**
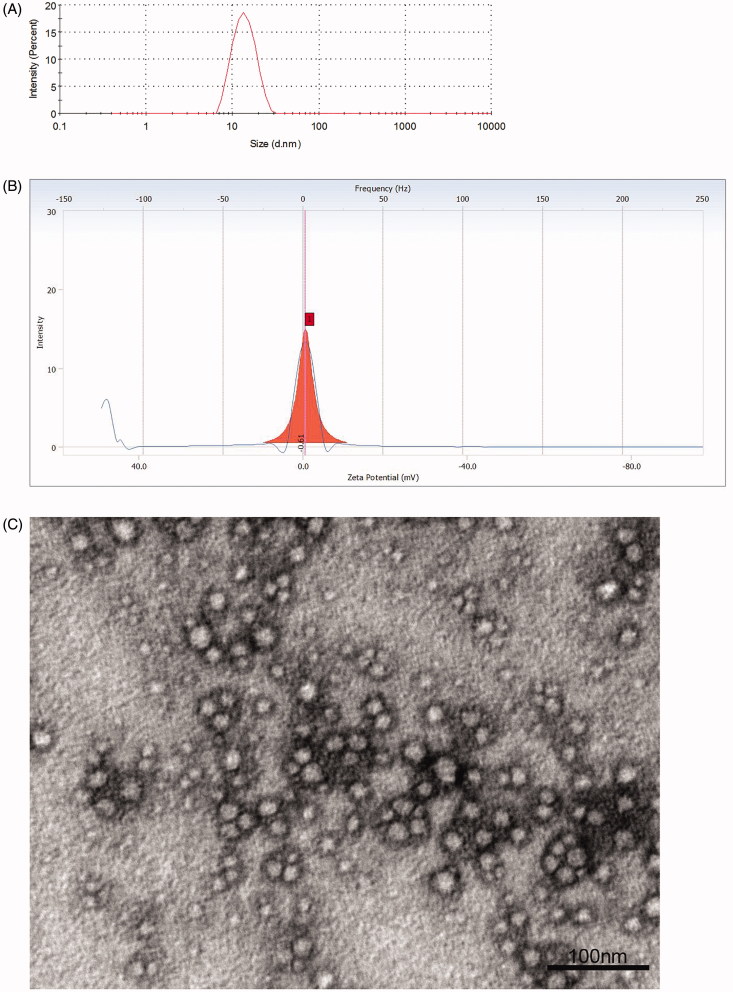
Characterization of the prepared LME formulations. (A) Representative graph of droplet size distribution for LME 5 (optimized LME); (B) representative graph of zeta potential distribution for LME 5 (optimized LME); (C) TEM image of LME 5 (optimized LME). The scale bar for image represents 100 nm. LME: lycopene-loaded microemulsion; TEM: transmission electron microscopy.

The results of average droplet size and PDI measurements are summarized in Table 3, and the representative graph is depicted in Figure 2(A). The average droplet size determined by DLS was in the range of 12.27 − 37.66 nm for these formulations, which were all less than 100 nm. More importantly, there existed significant difference in PDI analysis (one-way ANOVA, *p* < .01), and Bonferroni’s post-hoc tests revealed that the PDI values of LME 1 and 4 were both significantly larger than the remaining four formulations (LME 2, 3, 5 and 6) (*p*s <.01), with values >0.3 indicating heterogeneous dispersions (Parikh et al., 2017). Nevertheless, no remarkable difference was discovered in multiple comparisons for LME 2, 3, 5 and 6 (*p*s > .05, Bonferroni correction), with PDI values all <0.1 designating homogeneous dispersions (Parikh et al., 2017). Thus, the LME 1 and 4 were excluded from further optimization.

In terms of zeta potential, the values of the remaining LME 2, 3, 5 and 6 were all slight negative and/or close to neutral (Table 3, Figure 2(B)), and their difference was negligible (one-way ANOVA, *p* = .463).

As shown in Table 3, the lycopene content determined in samples of LME 2, 3, 5 and 6 ranged from 173.44 to 463.03 µg/mL, and the analysis reached significant level (one-way ANOVA, *p* < .01). Considering the need of animal studies and oral administration, the relative ratios of lycopene content to S_mix_ content were calculated for all remaining formulations in order to simultaneously increase drug content and decrease surfactant content, and the results were in the rank order of LME 5 > LME 6 > LME 2 > LME 3. Hence, LME 5 was regarded as the optimized formulation.

The representative TEM image of LME 5 (Figure 2(C)) demonstrated spherical and uniform shape of the nanoparticles with a small size, which was in agreement with the data of droplet size distribution measured using DLS analyzer.

Considering all the above-mentioned requirements, LME containing 8% w/w of lycopene and (*R*)-(+)-limonene, 32% w/w of Tween 80 and Transcutol HP (ratio = 2:1, w/w) and 60% w/w of water, respectively, was finalized. The optimized LME showed an average droplet size of 12.61 ± 0.46 nm with PDI value of 0.086 ± 0.028, and the spherical and homogeneous morphology was captured by TEM imaging. The zeta potential was determined to be −0.49 ± 0.12 mV. Furthermore, 463.03 ± 8.96 µg/mL lycopene was found to be soluble and could be incorporated into final ME system.

### 3.4. Stability study during storage

In this study, the optimized LME and LOO were stored at 4 and 25 °C to evaluate physical and chemical stabilities. Immediately after preparation and during storage at both 4 and 25 °C for a total of 8 weeks, LME was always transparent and there was no sign of precipitation or phase separation after centrifugation, indicating that the optimized LME demonstrated superior thermodynamic stability under long-term storage and accelerated condition (centrifugation). As for chemical stability, overall, the average percentages of lycopene remaining were significantly higher for LME compared with LOO after 2, 4, 6 and 8 weeks of storage (all *p* < .05 by two-way ANOVA), and subsequent post-hoc analysis revealed that these phenomena existed when they were placed at both 4 and 25 °C (see Table 4). After storage for 6 and 8 weeks, we observed higher percentages of lycopene remaining at 4 °C than those at 25 °C for LME (*p*s < .05). Therefore, compared with the conventional LOO dosage form, the optimized LME could better protect lycopene from degradation during storage, and this protective effect was more conspicuous when LME was stored at 4 °C.

**Table 4.** The chemical stability of different dosage forms during storage at 4 and 25 °C.

Dosage forms	Temperature (°C)	Percentages of lycopene remaining (%)					
		Baseline	1 week	2 weeks	4 weeks	6 weeks	8 weeks
LOO	4	100	92.25 ± 2.90	86.45 ± 3.81	82.83 ± 2.65	79.71 ± 2.36	75.76 ± 1.82
	25	100	91.85 ± 2.70	85.17 ± 3.93	78.10 ± 1.79	74.62 ± 1.31	70.14 ± 3.25
Optimized LME	4	100	96.03 ± 2.48	92.99 ± 2.82*	90.31 ± 3.20**	88.76 ± 1.96^**&^	87.04 ± 2.43^**&^
	25	100	95.11 ± 2.81	91.66 ± 2.69^#^	87.38 ± 2.01^##^	84.58 ± 1.74^##^	82.09 ± 2.60^##^

LOO: lycopene dissolved in olive oil; LME: lycopene-loaded microemulsion.

Each value is the mean ± SD of three separate determinations. Baseline represents day 0. Statistical significances were performed by two-way ANOVA (Bonferroni correction).

*p < .05, **p < .01 compared with LOO at 4 °C.

^#^*p* < .05, ^##^*p* < .01 compared with LOO at 25 °C.

& *p* < .05 compared with optimized LME at 25 °C.

### 3.5. Analytical method validation

Before the commencement of sample determinations, analytical method validation was conducted in rat plasma, as well as mouse plasma, brain and liver as representations. The precisions, accuracy, extraction recovery and stability are displayed in Table 5. By analyzing five replicates of quality control samples at three corresponding concentration levels (low, medium and high) on the same day and on three consecutive days, the obtained results of intra-day precision, inter-day precision and accuracy were all within the accepted variable limits, indicating that the method was precise and accurate for the determination of lycopene in plasma and tissues. When the external standard method was used, the extraction recoveries of lycopene in different samples ranged from 80.53 to 86.83%, with all relative standard deviations (RSDs) less than 8.35%. Moreover, by employing retinyl acetate as the internal standard (Miller et al., 1984; Milne & Botnen, 1986), similar values were obtained for extraction recoveries of lycopene, which also fulfilled the requirements of bio-sample determinations. These results all suggested that the method used for analysis was satisfactory. In addition, the quality control samples were also found to be stable after being placed at room temperature (25 °C) for 4 h and stored at −80 °C for 6 weeks, respectively.

**Table 5.** Precisions, accuracy, extraction recovery and stability for the determination of lycopene in different samples.

Samples	Concentrations	Intra-day precision (*n* = 5)	Inter-day precision (*n* = 3)	Accuracy	Extraction recovery (*n* = 5)				Stability (*n* = 5)	
					With external standard^a^		With internal standard^b^		25 °C(4 h)^c^	−80 °C (6 weeks)^d^
		RSD (%)	RSD (%)	RE (%)	Mean (%)	RSD (%)	Mean (%)	RSD (%)	RE (%)	RE (%)
Rat plasma	10 ng/mL	3.67	8.35	3.77	83.80	5.47	80.66	7.43	−6.72	−5.00
	80 ng/mL	6.08	7.53	−1.65	82.65	6.18	85.06	3.44	1.08	−1.05
	320 ng/mL	4.02	2.85	−1.08	82.90	4.44	87.82	4.02	−5.19	−2.60
Mouse plasma	10 ng/mL	14.30	8.29	5.90	86.83	5.11	83.47	3.71	−2.69	−4.44
	80 ng/mL	8.02	5.35	1.19	80.71	7.02	82.02	2.40	−2.85	−4.38
	320 ng/mL	9.46	1.42	−8.74	82.09	8.35	82.43	3.05	−1.29	−4.59
Mouse brain	10 ng/g	7.73	5.76	−4.85	80.53	6.95	80.05	7.43	7.30	−10.93
	40 ng/g	10.77	3.15	3.55	82.20	4.86	83.75	8.07	3.58	−1.75
	160 ng/g	6.07	6.61	1.16	81.90	3.88	86.05	9.52	5.91	3.92
Mouse liver	10 ng/g	3.26	3.62	−4.11	81.99	2.22	85.62	4.91	−4.89	−3.94
	80 ng/g	3.78	2.04	−1.01	86.68	2.65	85.26	5.21	−1.67	−3.35
	320 ng/g	2.59	4.50	−1.02	82.47	1.98	89.96	7.22	−3.98	−4.32

RE: relative error; RSD: relative standard deviation.

a Lycopene as the external standard.

b Retinyl acetate as the internal standard.

c The quality control samples were placed at room temperature (25 °C) for 4 h and were protected from light exposure.

d The quality control samples were stored at −80 °C for 6 weeks. They were all protected from light exposure and flushed with nitrogen gas.

### 3.6. Pharmacokinetic study in rats

**Figure 3.**
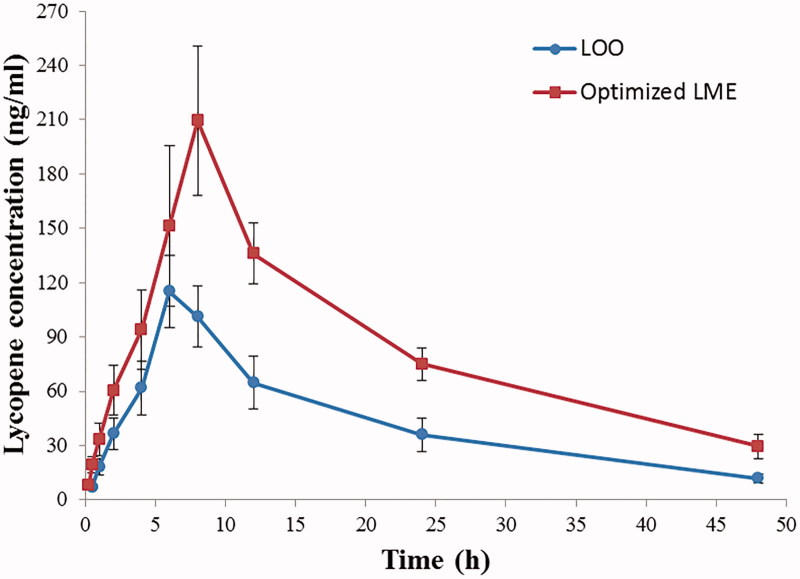
Plasma concentration–time profiles of lycopene in rats after oral administration of LOO and optimized LME. Each data point represents the mean ± SD of six determinations. LOO: lycopene dissolved in olive oil; LME: lycopene-loaded microemulsion.

Figure 3 depicts plasma concentration–time profiles of lycopene after oral administration of two formulations to rats, and the pharmacokinetic parameters are summarized in Table 6. The AUC_(0–∞)_ of lycopene in the test group (optimized LME) (4775.93 ± 634.00 h·ng/mL) was significantly increased compared with that in the control group (LOO) (2270.96 ± 455.46 h·ng/mL, *p* < .01), and the relative bioavailability was elevated to 210.30%, indicating a dramatic enhancement in absorption and oral bioavailability of lycopene. Besides, the *C*_max_ value for LME was 220.48 ± 30.84 ng/mL, which was 1.82 times greater than that for LOO. Furthermore, enhanced *t*_1/2_, longer MRT_(0–∞)_ and lower CL were observed in LME-treated rats, suggesting a prolonged residence time and slower elimination. The *T*_max_ did not differ significantly between two groups. These results demonstrated an increased drug exposure in blood circulation for optimized LME.

**Table 6.** Pharmacokinetic parameters of lycopene in rat plasma following oral administration.

Parameters	LOO	Optimized LME
AUC_(0–∞)_ (h·ng/mL)	2270.96 ± 455.46	4775.93 ± 634.00**
*C*_max_ (ng/mL)	121.32 ± 13.47	220.48 ± 30.84**
*T*_max_ (h)	6.33 ± 0.82	7.67 ± 0.82
*t*_1/2_ (h)	13.75 ± 1.10	17.01**±**2.61*
MRT_(0–∞)_ (h)	21.49 ± 1.11	25.94**±**3.70*
CL (L/h/kg)	3.64 ± 0.74	1.70 ± 0.23**
Relative bioavailability^a^	–	210.30%

LOO: lycopene dissolved in olive oil; LME: lycopene-loaded microemulsion; AUC: area under the concentration-time curve; *C*_max_: peak concentration; *T*_max_: time to reach peak concentration; *t*_1/2_: half-life; MRT: mean residence time; CL: plasma clearance.

Each value is the mean ± SD of six rats. Statistical significances were performed as follows: *t*_1/2_, MRT and CL: one-way ANOVA; AUC and *C*_max_: one-way ANOVA following logarithmic transformation; *T*_max_: Mann–Whitney U-test.

*p < .05, **p < .01 compared with LOO.

a LOO as reference.

### 3.7. Tissue distribution study in mice

The standard curves and linear ranges of lycopene in mouse plasma and tissues are plotted and presented in Supplementary Table S2. All standard calibrations exhibited good linearity and satisfactory correlation coefficients.

**Figure 4.**
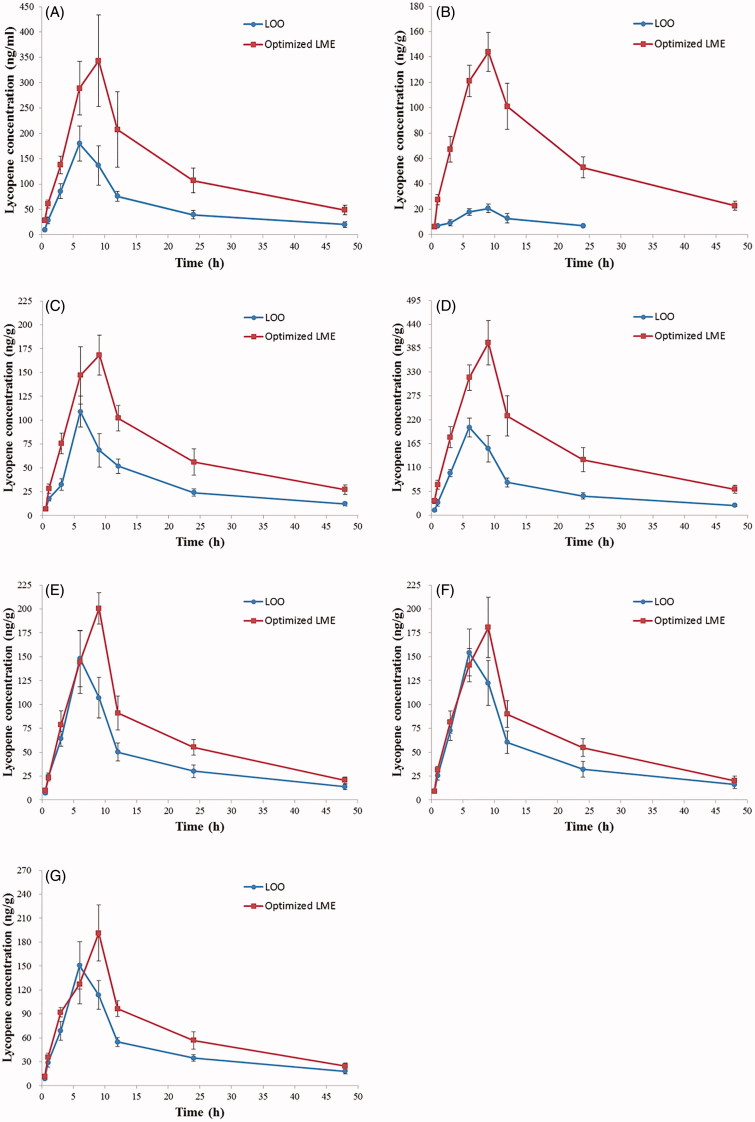
Concentration–time profiles of lycopene in various tissues of mice after oral administration of LOO and optimized LME. (A) Plasma; (B) brain; (C) heart; (D) liver; (E) spleen; (F) lung; (G) kidney. Each data point represents the mean ± SD of six determinations. LOO: lycopene dissolved in olive oil; LME: lycopene-loaded microemulsion.

The concentration–time profiles of lycopene in mouse tissues can be seen in Figure 4(A–G), respectively, from which various pharmacokinetic and targeting parameters were calculated as displayed in Table 7.

**Table 7.** Pharmacokinetic and targeting parameters of lycopene in mouse tissues following oral administration (*n* = 6).

Tissues	Dosage forms	AUC_(0–∞)_ (h·ng/mL)/(h·ng/g)	*C*_max_ (ng/mL)/(ng/g)	*T*_max_ (h)	*t*_1/2_ (h)	MRT_(0–∞)_ (h)	*R*e	*C*e	DTI
Plasma	LOO	2792.87	179.99 ± 34.42	6	8.29	20.65			
	Optimized LME	6889.13	343.30 ± 90.14**	9	12.57	24.31			
Brain	LOO	416.81	20.62 ± 3.39	9	13.27	21.10			
	Optimized LME	3549.52	143.86 ± 15.27**	9	17.19	26.15	8.52	6.98	3.45
Heart	LOO	1671.06	108.91 ± 16.27	6	10.18	22.33			
	Optimized LME	3563.70	168.29 ± 21.14**	9	13.05	25.58	2.13	1.55	0.86
Liver	LOO	3058.30	202.22 ± 22.09	6	8.28	20.83			
	Optimized LME	8192.24	397.77 ± 51.03**	9	13.82	25.52	2.68	1.97	1.09
Spleen	LOO	2089.52	148.14 ± 29.51	6	7.98	19.68			
	Optimized LME	3685.29	200.51 ± 16.46**	9	16.81	24.11	1.76	1.35	0.72
Lung	LOO	2318.79	154.37 ± 24.43	6	7.90	20.03			
	Optimized LME	3605.43	180.77 ± 31.54	9	16.72	24.06	1.55	1.17	0.63
Kidney	LOO	2316.29	150.65 ± 29.78	6	8.57	21.69			
	Optimized LME	3704.39	191.43 ± 34.88	9	15.49	25.37	1.60	1.27	0.65

LOO: lycopene dissolved in olive oil; LME: lycopene-loaded microemulsion; AUC: area under the concentration-time curve; *C*_max_: peak concentration; *T*_max_: time to reach peak concentration; *t*_1/2_: half-life; MRT: mean residence time; *R*e: the relative rates of uptake; *C*e: the ratio of peak concentration; DTI: drug targeting index.

Statistical significances were performed as follows: *C*_max_: one-way ANOVA following logarithmic transformation.

** *p* < .01 compared with LOO.

As depicted in Figure 4(A), after oral delivery of LME, the plasma concentrations of lycopene were significantly higher than those following LOO administration at all-time points (0.5, 1, 3, 6, 9, 12, 24, 48 h; *p*s < .01), as well as for *C*_max_ (LOO: 179.99 ± 34.42 ng/mL, optimized LME: 343.30 ± 90.14 ng/mL; *p* < .01). There is a 2.47-fold enhancement of AUC_(0–∞)_ value with optimized LME (6889.13 h·ng/mL) than LOO (2792.87 h·ng/mL), implying an increased distribution of lycopene in mouse plasma. Additionally, the *T*_max_ was relatively delayed in LME compared to LOO.

Following oral administration, the *C*_max_ values of brain, heart, liver and spleen were markedly increased in the test group (optimized LME) in comparison to those in the control group (LOO) (all *p* < .01), while for lung and kidney tissues, the differences did not reach significant levels (*p*s > .05). Compared with LOO, the biodistributions of lycopene were greatly altered for optimized LME, with longer *t*_1/2_ and MRT_(0–∞)_ in all tested tissues (Table 7).

Figure 4(B) and Table 7 show the mean concentration versus time profiles of brain and corresponding pharmacokinetic parameters, respectively. In addition to remarkably enhanced *C*_max_ (20.62 ± 3.39 ng/g for LOO and 143.86 ± 15.27 ng/g for optimized LME, *p* < .01), a surprising 8.52-fold higher AUC_(0–∞)_ value was found in LME-treated mouse brain (3549.52 h·ng/g) with respect to that administered with LOO (416.81 h·ng/g). We also discovered comparatively longer *t*_1/2_ and MRT_(0–∞)_ for LME, and lycopene concentration was undetectable in brain tissue 48 h after LOO delivery, whereas for optimized LME, it could still be detected at 48 h post-administration (22.79 ± 3.60 ng/g), which further confirmed slower clearance and prolonged retention of lycopene in the brain provided by LME. Taken together, the aforementioned results indicated that the optimized LME distinctly facilitated brain uptake of lycopene.

### 3.8. Drug targeting evaluation

As presented in Table 7, the *C*e values of brain, heart, liver, spleen, lung and kidney were calculated to be 6.98, 1.55, 1.97, 1.35, 1.17 and 1.27, respectively, with the maximum obtained for brain tissue. In terms of the *R*e parameter, it was greatest for the brain (8.52), followed by the liver (2.68), heart (2.13), spleen (1.76), kidney (1.60) and lung tissues (1.55), proving a dramatic increase of lycopene distribution in the brain.

More importantly, the parameter of DTI was implemented for the purpose of better evaluating blood-to-tissue direct transport and targeting efficiency. The DTI values were determined to be less than 1 for the heart (0.86), spleen (0.72), kidney (0.65) and lung tissues (0.63) and slightly more than 1 for the liver (1.09). Nevertheless, with regard to the brain tissue, this value was much higher (3.45), suggesting a preferential targeting distribution toward brain for optimized LME in comparison with the conventional LOO dosage form.

## 4. Discussion

In the current study, a novel LME system composed of lycopene and (*R*)-(+)-limonene (oil), Tween 80 (surfactant), Transcutol HP (co-surfactant) as well as water was successfully prepared and further characterized for stability, droplet size distribution, zeta potential, lycopene solubilization capacity and morphological assessment. The optimized LME demonstrated small droplet size with narrow size distribution, and the spherical and uniform shape was observed by TEM imaging, implying that the homogeneous dispersion was obtained. It also possesses excellent physical and chemical stabilities. In addition, upon oral delivery, LME showed a 2.10-fold increase of relative bioavailability compared with LOO in rats. Notably, this new formulation prolonged residence time, delayed elimination, together with dramatically enhancing the distribution of lycopene in the brain (DTI = 3.45) in mice, indicating the superiority of optimized LME in enabling targeted brain delivery of lycopene following oral administration.

In terms of screenings of ME excipients, the orthogonal design was employed for the purpose of better assessing constituent interactions and reducing experimentations (9 tests, see Figure 1) (Cai et al., 2012; Cao et al., 2017). The construction of pseudo-ternary phase diagrams, therefore, was aimed at determining the appropriate proportion of compositions for ME system (Syed & Peh, 2014), and the impacts of these factors (oil, co-surfactant and surfactant to co-surfactant ratio) on ME formation were evaluated by areas of ME region, since a stable and broad region could maintain the physicochemical properties of ME through the drug absorption period in the gastrointestinal tract. Therefore, this criterion is necessary for formulation selection (Subongkot & Ngawhirunpat, 2017). The selection of oil phase based on solubility study is critical to prevent drug precipitation during storage (Parikh et al., 2017), and higher solubility could increase drug incorporation, facilitate absorption and provide better protection against undesired degradations (Gupta et al., 2013). In our previous experiments, we performed preliminary selection of oil phase from various candidates according to their lycopene solubility. After orthogonal optimization, (*R*)-(+)-limonene, with the highest lycopene solubility, was chosen as the optimum oil, and it has also been applied in several earlier researches (Spernath et al., 2002; Liu et al., 2011).

As a solubilizer, stabilizer and permeation enhancer, the surfactant in lipid-based nanoformulations plays important roles in regulating *in vivo* pharmacokinetics of drugs (Parikh et al., 2017). Tween 80 (HLB = 15) is a nonionic surfactant owning excellent emulsifying capability, and is biocompatible with a wide range of hydrophobic drugs. This commercially available emulsifier is considered to be nontoxic and nonirritant for oral administration (Rowe et al., 2009). Additionally, Tween 80 could facilitate oral drug bioavailability (Sangsen et al., 2016) and brain-targeted pharmaceutical delivery (Sun et al., 2004; Craparo et al., 2008; Wilson et al., 2008). So Tween 80 was selected as the desirable surfactant. Another essential ingredient the co-surfactant, serving as a vehicle of ME system, helps to lower the interfacial tension and increase the fluidity of interfacial membrane around these nanoparticles (Kawakami et al., 2002b). The results of orthogonal optimization confirmed the candidature of Transcutol HP as a co-surfactant. With good biocompatibility and proven safety profile (Rowe et al., 2009), this co-emulsifier has been commonly used in the construction of orally administered ME formulations (Wu et al., 2015; Guo et al., 2016). Furthermore, the surfactant to co-surfactant weight ratio is a key factor affecting S_mix_ interactions and ME formation (Chen et al., 2017), and the appropriate ratio was fixed at 2:1.

Several LME formulations were prepared using the water titration method, of which LME 5 fulfilled the selection criteria of good thermodynamic stability, satisfactory droplet size, low PDI value, together with high lycopene incorporation content and relatively low S_mix_ content (Yeom et al., 2015), so it was chosen as the optimized formulation. The average droplet size is a crucial parameter influencing pharmaceutical characteristics and biodistribution of preparations. Besides, the measurement of PDI is employed to understand the range of droplet size in ME system, and its value closer to zero suggests greater uniformity of the formed dispersion. It has been reported that nanoscale-sized particles with homogeneous distribution can provide a large surface area, improve drug absorption (Mohsin et al., 2016), as well as making it easier to cross the BBB (Sun et al., 2015). Therefore, the optimized LME was a monodispersed system and appropriate for delivery.

Zeta potential is an indispensable property of the formed dispersion, and the larger negative zeta potential of nanoparticles was an important factor for its physical stability (Chansiri et al., 1999). In the present study, the slight negative zeta potential value of −0.49 ± 0.12 mV for optimized LME could be attributed to nonionic nature of surfactant and co-surfactant. However, the optimized formulation demonstrated good stability during the short-term and long-term storage, together with centrifugal tests. Similarly, a few earlier researches showed that although their prepared microemulsions had very low zeta potentials, these samples were found stable after several months of storage (Acharya et al., 2013; Subongkot & Ngawhirunpat, 2017). Our optimized LME was stable even at low zeta potential perhaps due to the extremely small droplet size and narrow size distribution (Tao et al., 2017).

There have been several investigations indicating low oral bioavailability of lycopene (Tang et al., 2005; Faisal et al., 2010). In pharmacokinetic study, using LOO as the control, we discovered that the relevant parameters were greatly changed for optimized LME in rats, including significantly increased AUC_(0–∞)_ and *C*_max_, remarkably prolonged *t*_1/2_, MRT_(0–∞)_ and lower CL. The dramatic 2.10-fold improvement of relative bioavailability was observed for optimized LME, which could be explained by the combination of the following effects: (1) The larger surface area provided by small droplet size and narrow size distribution of optimized LME allows pharmaceuticals to better interact with gastrointestinal mucosa, which could increase the rate of drug absorption (Mohsin et al., 2016). (2) The ME system might facilitate intestinal cellular uptake and lymphatic transport, which could contribute to the enhancement of lycopene absorption (Tang et al., 2013; Bala et al., 2016). (3) Tween 80 and Transcutol HP could exert synergistically as inhibitors of P-glycoprotein multidrug efflux system (Takahashi et al., 2002; Sun et al., 2016), which is mainly localized in the columnar epithelial cells of the lower gastrointestinal tract (Zakeri-Milani & Valizadeh, 2014). Thus, these microemlusion excipients could potentially enhance oral bioavailability of lycopene. (4) The improvement of oral bioavailability mediated by optimized LME might also be due to smaller nanoparticles transported in blood circulation, which make them harder to be taken up by phagocytosis (des Rieux et al., 2006).

As for the tissue distribution study in mice, enhanced lycopene biodistributions were obtained for optimized LME compared with the conventional LOO in all tissues. Among the tested tissues, the values of *R*e and *C*e parameters were all exceeding 1, of which brain displayed the largest values (8.52 and 6.98, respectively). Considering oral administration, the DTI parameter was adopted to assess tissue targeting efficiency. The DTI values of spleen (0.72) and lung (0.63) were less than 1, suggesting that the ME nanoparticles were less likely to be captured by the reticuloendothelial system. The kidney tissue exhibited a low value (0.65) as well, which was possibly due to the attenuated renal excretion of lycopene. Most importantly, the DTI value of brain was up to 3.45, implying superior brain-targeting capability for optimized LME.

To deliver therapeutic levels of drugs for treatment of brain-related ailments remains a major challenge owing to presence of the protective BBB, which forms an obstacle and prohibits entry for a range of neuropharmaceuticals into brain parenchyma (Henderson & Piquette-Miller, 2015). Nevertheless, lycopene, regarded as a promising neuroprotector, was transported through the BBB more efficiently when incorporated into ME system, which might be attributed to the following causes: (1) Tween 80 could mediate the endocytosis of ME nanoparticles by the endothelial cells lining the brain blood capillaries, which leads to release of lycopene within these cells and further delivery to brain parenchyma (Kreuter, 2001). (2) Tween 80 could adsorb apolipoprotein E (apo E) from systemic circulation onto the surface of ME nanoparticles, then apo E interacts with the brain low-density lipoprotein receptors, which exist in the BBB (Meresse et al., 1989). Afterwards, the nanoparticles could be uptaken by the brain capillary endothelial cells via the mechanism of receptor-mediated endocytosis (Prabhakar et al., 2013). (3) In addition to the gut, the P-glycoprotein multidrug efflux pump is also localized in the brain capillary endothelial cells (Schinkel, 1999), and its function could be suppressed by Tween 80 and Transcutol HP (Takahashi et al., 2002; Sun et al., 2015), which might contribute to improvement in targeting distribution of lycopene into the brain. (4) The smaller droplet size of optimized LME might potentially enhance permeation of lycopene across the BBB (Shah et al., 2016). (5) The prolonged retention of LME in the blood could create a higher concentration gradient in brain capillaries, thus facilitating transport across the endothelial cell layer and leading to lycopene accumulation in the brain (Ma et al., 2013). Altogether, the combined effects described above might result in the dramatically enhanced targeting distribution of lycopene in brain tissue mediated by optimized LME, while the exact underlying mechanisms required further elucidation in our future work.

In terms of the previous reports on targeted delivery of drugs to the brain, nose to brain delivery with microemulsions and other systems is a most investigated and efficient route (Shah et al., 2016; Salem et al., 2019). In addition, there have been several investigations available at present implying that oral delivery of ME preparations with specific compositions could also promote targeting distribution of pharmaceuticals in the brain (Wang et al., 2012; Ma et al., 2013). In this study, Tween 80 was selected as the surfactant for the prepared LME formulation, which could facilitate brain-targeted delivery of nanoparticles (Kreuter, 2001; Sun et al., 2004). Our findings indicated that, in comparison with the conventional LOO dosage form, the optimized LME system could improve intestinal absorption and oral bioavailability of lycopene, as well as enhancing subsequent blood to brain targeting transport (DTI = 3.45). As a consequence, the lycopene concentration was greatly elevated in brain tissue, demonstrating that LME possessed good brain-targeting capability.

As for the transport of lycopene after intestinal absorption, the control LOO dosage form could be converted to chylomicrons, which were further transported and metabolized in the systemic circulation, while LME might also participate in the formation of chylomicrons during absorption. Nevertheless, as reflected by the DTI parameter, the optimized LME dramatically enhanced blood to brain targeting delivery of lycopene when compared with LOO (DTI = 3.45), suggesting that the transport and metabolisms of LME could be different from those of LOO dosage form. This might be due to the specific structures and constituents of LME, such as the surfactant Tween 80 and the co-surfactant Transcutol HP, while the exact mechanisms need to be further investigated.

## 5. Conclusions

In this investigation, we reported the development and characterization of a ME system incorporated with lycopene. The optimized LME formulation consisting of lycopene and (*R*)-(+)-limonene as the oil phase, Tween 80 and Trancutol HP as the S_mix_ and water was selected, and its physicochemical parameters were found to be satisfactory, including high lycopene incorporation content, small droplet size with narrow size range, spherical ultrastructural morphology and good *in vitro* stability. Furthermore, in comparison with the conventional dosage form (LOO), this new ME showed improvement of oral bioavailability in rats and superior brain-targeting efficiency in mice, which were both the first time for LME delivery. Given this, the ME drug delivery system could possibly not only facilitate the therapeutic effect of lycopene, particularly for neurological disorders, but also be employed as a promising and versatile nanocarrier for targeted brain delivery of many other poorly water-soluble pharmaceuticals by oral administration.

## Supplementary Material

Supplemental Material

Supplemental Material
