# Supplementary material for: Oral delivery of lycopene-loaded microemulsion for brain-targeting: preparation, characterization, pharmacokinetic evaluation and tissue distribution
Source: Drug Deliv. 2019 Nov 18;26(1):1191–205. doi: 10.1080/10717544.2019.1689312 (PMC6882477; doi:10.1080/10717544.2019.1689312)
Supplement: Supplemental Material [file IDRD_A_1689312_SM5827.docx]

**Table S2.** The standard curves for lycopene in plasma and tissues

| Animals | Samples | Linear range (ng/g) | Regression equation | Coefficient R |
| --- | --- | --- | --- | --- |
| Rat | Plasma^a^ | 5−640 | *A* = 80.1400*C* − 47.5278 | 0.9990 |
| Mouse | Plasma^a^ | 5−640 | *A* = 71.0717*C* + 4.0674 | 0.9958 |
|  | Brain | 5−320 | *A* = 152.0399*C* − 97.9559 | 0.9978 |
|  | Liver | 5−640 | *A* = 169.2794*C* – 157.6984 | 0.9985 |
|  | Kidney | 5−640 | *A* = 164.7473*C* – 195.3909 | 0.9989 |
|  | Heart | 5−640 | *A* = 66.6174*C* + 8.2678 | 0.9989 |
|  | Lung | 5−640 | *A* = 68.3617*C* + 18.9422 | 0.9972 |
|  | Spleen | 5−640 | *A* = 67.8817*C* + 57.3532 | 0.9961 |

*A* is the peak area of lycopene in plasma or tissues; *C* is the concentration of lycopene standards.

^a^ The concentration in plasma is given as ng/mL.
