# Supplementary material for: Oral delivery of lycopene-loaded microemulsion for brain-targeting: preparation, characterization, pharmacokinetic evaluation and tissue distribution
Source: Drug Deliv. 2019 Nov 18;26(1):1191–205. doi: 10.1080/10717544.2019.1689312 (PMC6882477; doi:10.1080/10717544.2019.1689312)
Supplement: Supplemental Material [file IDRD_A_1689312_SM5825.docx]

**Table S1.** Factors and levels of orthogonal optimizing experiments

|  |  | Factors |  |
| --- | --- | --- | --- |
| Levels | A (oil) | B (co-surfactant) | C (surfactant/co-surfactant) |
| 1 | (*R*)-(+)-Limonene | Transcutol HP | 2:1 |
| 2 | Ethyl oleate | PEG 400 | 3:2 |
| 3 | Oleic acid | Glycerol | 3:1 |

Surfactant/co-surfactant denotes the surfactant to co-surfactant ratio (w/w).

PEG 400: polyethylene glycol 400.
